# Application of Chitinous Materials in Production and Purification of a Poly(l-lactic acid) Depolymerase from *Pseudomonas tamsuii* TKU015

**DOI:** 10.3390/polym8030098

**Published:** 2016-03-22

**Authors:** Tzu-Wen Liang, Shan-Ni Jen, Anh Dzung Nguyen, San-Lang Wang

**Affiliations:** 1 Department of Chemistry, Tamkang University, New Taipei City 25137, Taiwan; ltw27@ms55.hinet.net (T.-W.L.); wennysunny@yahoo.com.tw (S.-N.J.); 2 Life Science Development Center, Tamkang University, New Taipei City 25137, Taiwan; 3 Biotechnology Centre, Tay Nguyen University, 567 Le Duan Str., Buon Ma Thuot 567, Vietnam; nadzungtaynguyenuni@yahoo.com.vn

**Keywords:** PLA, squid pen, *Pseudomonas tamsuii*, PLA depolymerase, degradation

## Abstract

The management of fishery residues and plastics is considered to be a vital strategy for conserving resources and maintaining the quality of the environment. Poly(l-lactic acid) (PLA) is a commercially promising, renewable, and biodegradable plastic. In this study, a PLA depolymerase was produced in a squid pen powder (SPP) and recycled plastic waste (PLA powder)-containing medium by *Pseudomonas tamsuii* TKU015, a bacterial strain isolated from Taiwanese soil. This PLA depolymerase had a molecular weight of 58 kDa and was purified to homogeneity from the supernatant of a TKU015 culture. The optimum pH of TKU015 PLA depolymerase is 10, and the optimal temperature of the enzyme is 60 °C. In addition to PLA, TKU015 PLA depolymerase degraded fibrinogen and tributyrin, but did not hydrolyze casein, triolein, and poly(β-hydroxybutyrate). Taken together, these data demonstrate that *P. tamsuii* TKU015 produces a PLA depolymerase to utilize SPP and polylactide as carbon/nitrogen sources.

## 1. Introduction

Biodegradable plastics (BPs) can be degraded into water and CO_2_ by microorganisms in bioactive environments. Thus, from the perspective of environmental protection and solid waste management, BPs are increasingly considered as attractive alternatives to petroleum-derived plastics. Poly(l-lactic acid) (PLA) is a commercially promising, renewable, and biodegradable plastic. However, the degradation of PLA in natural environments is more difficult than other aliphatic polyesters such as poly(β-hydroxybutyrate) (PHB) and poly(ε-caprolactone) (PCL) [1]. Therefore, methods for enhanced degradation of PLA must be developed. Treatment with PLA-degrading enzymes is one such method that is expected to accelerate PLA degradation. Recently published works have reported that PLA is hydrolysable by enzymes such as proteinase K from *Tritirachium album* [2,3,4], a silk fibroin-degrading enzyme from *Amycolatopsis orientalis* [5], lipases from *Rhizopus delemer* [4] and *Alcaligenes* sp. [6], esterase from *Paenibacillus amylolyticus* TB-13 [7], cutinase-like enzyme from *Cryptococcus* sp. S-2 [8], and the polyester polyurethane-degrading enzyme from *Comamonas acidovorans* TB-35 [9]. Most of the reported PLA-degrading enzymes belong to the serine protease family, as PLA has as structure similar to fibroin, a native substrate of serine proteases [10]. However, because PLA is an aliphatic polyester, it can also be decomposed by lipases or esterases. Most of the PLA-degrading strains belong to the *Amycolatopsis* genus [11,12,13,14], a genus that might play an important role in the natural biodegradation of PLA [15]. However, it is unclear as to how these various enzymes with different catalytic properties achieve the hydrolysis of PLA. It is also unclear whether several enzymes are needed simultaneously to completely degrade PLA. Furthermore, it is interesting to find other more unique enzymes capable of efficiently degrading PLA.

In this study, a PLA-degrading bacterium, *Pseudomonas tamsuii*, was isolated. This bacterium exhibited a significantly high level of PLA-degrading activity when squid pen powder and recycled plastic waste (PLA powder) were added to the basal liquid medium. The use of inexpensive culture media as well as practical fermentation processes can produce PLA-degrading enzymes for large-scale recycling of PLA wastes in an economically efficient manner. The production of this enzyme and its use to recycle fishery wastes and PLA has not been reported previously. In addition, a PLA-degrading enzyme was purified from *Pseudomonas tamsuii* TKU015.

## 2. Experimental

### 2.1. Materials

Shrimp shells and squid pens were purchased from Shin-Ma Frozen Food Co. (I-Lan, Taiwan) and used as carbon/nitrogen sources for the production of PLA-degrading enzymes by microorganisms. Recycled PLA plastic wastes were prepared as powders and added to the basal liquid medium for the production of PLA-degrading enzymes. PLA pellets (2002D, number-average molecular weight, *M*_n_: 1.25 × 10^4^) were obtained from Cargill Dow, Minnetonka, MN, USA. PLA powders (particle size 200–300 μm) for enzymatic assays were prepared by dissolving 50 mg of PLA pellets in 50 mL of dichloromethane mixed with 500 mL of methanol. PLA powders were obtained by homogenizing the PLA/dichloromethane/methanol solution with a homogenizer at 10,000 rpm for 5 min. PLA powders were filtered and air-dried for 24 h, then dried in a vacuum oven overnight at 30 °C. All chemicals used were of analytical grade.

### 2.2. Strains

The strains used in this study include *Pseudomonas tamsuii* TKU015, which was isolated from Taiwanese soil and stocked in our laboratory [16]. *P. tamsuii* TKU015 produced high levels of extracellular chitinase, chitosanase, nattokinase, and insecticidal activities when grown with shrimp shell powder (SSP) or squid pen powder (SPP) as the sole carbon and nitrogen source [16,17,18]. The strain was maintained on SPP plates (0.5% (*w*/*v*) SPP, 0.1% (*w*/*v*) K_2_HPO_4_, 0.05% (*w*/*v*) MgSO_4_·7H_2_O, and 1.5% (*w*/*v*) agar) at 4°C.

### 2.3. Screening of PLA-Degrading Microorganisms

Microorganisms were screened for PLA degradation activity in a medium containing 0.25% (*w*/*v*) recycled plastic wastes (PLA powder), 0.25% (*w*/*v*) SPP, 0.1% (*w*/*v*) K_2_HPO_4_, 0.05% (*w*/*v*) MgSO_4_·7H_2_O, and 1.5% (*w*/*v*) agar in distilled water. This medium was also used for routine cultivation of the screened microorganism. PLA-degrading strains were screened based on growth on PLA/SPP agar medium at 30°C for 5–10 days and to formation of clear zones around colonies. Organisms obtained from this screening process were subcultured in liquid media (containing 0.25% SPP, 0.25% PLA powder, 0.1% K_2_HPO_4_ and 0.05% MgSO_4_·7H_2_O) in shaking flasks at 30 °C on a rotary shaker (150 rpm, LM-570R, Yihder, New Taipei City, Taiwan). After incubation for five days, the culture broth was centrifuged (4 °C at 12,000× *g* for 20 min, Model 5922, Kubota, Tokyo, Japan), and supernatants were collected for PLA-degrading activity measurements using the procedure described below. *P. tamsuii* TKU015 showed the highest PLA-degrading activity. This organism was maintained on PLA/SPP agar and used throughout this study.

### 2.4. Investigation of Flask Culture Conditions

The strain was pre-cultivated in a flask containing 0.25% (*w*/*v*) recycled plastic wastes (PLA powder), 0.25% (*w*/*v*) SPP, 0.1% (*w*/*v*) K_2_HPO_4_, and 0.05% (*w*/*v*) MgSO_4_·7H_2_O at 30 °C with reciprocal shaking at 150 rpm for 24 h. This medium, which contained minimal concentrations of PLA powder and SPP, was named MPS. One millilitre of pre-culture was transferred into 100 mL of PLA/SPP medium with appropriate concentrations of PLA and SPP. Three types of enriched MPS (2×MPS, 3×MPS and 4×MPS) contained two, three, and four times the aforementioned amounts of PLA powder and SPP in MPS. The culture temperature, volume and pH of the medium were also investigated. Cultures were cultivated under optimal culture conditions with rotary shaking at 150 rpm for five days. At various times during the cultivation, 1-mL aliquots of the culture were harvested, and the optical densities at 660 nm (OD_660_) of the aliquots were measured to investigate cell growth. In parallel, the culture broth was centrifuged (4 °C at 12,000× *g* for 20 min), and the enzymatic activities of the supernatant were measured as described below.

### 2.5. PLA-Degrading Activity Assays

PLA powder (0.1%, *w*/*v*) was emulsified with Plysurf A210G (0.01%, *w*/*v*) in 50 mM phosphate buffer (pH 7.0) and used as the substrate. Enzyme solutions were mixed with PLA emulsions to various final concentrations in a total volume of 2 mL, then incubated at 50 °C for 3 h. The degradation products that were formed were measured with an l-Lactic acid assay kit (Megazyme, Wicklow, Ireland). One unit (U) of PLA-degrading activity was defined as the amount of enzyme required to produce 1 μmol of lactate equivalent in 1 h.

### 2.6. Purification of PLA-Degrading Enzyme

The TKU015 strain was cultivated in medium containing recycled plastic wastes (PLA powder) and fishery wastes (SPP) under optimal culture conditions. After incubation, the culture medium (800 mL in total) was centrifuged at 12,000× *g* for 20 min, and ammonium sulphate (608 g/L) was added to the culture supernatant (720 mL in total). The resulting mixture was stored at 4 °C overnight, and the precipitate was collected by centrifugation at 4 °C for 20 min at 12,000× g. The precipitate was then dissolved in a small amount of 50 mM sodium phosphate buffer (pH 7) and dialyzed against the buffer. The resulting dialysate (crude enzyme, 35 mL in total) was loaded onto a DEAE-Sepharose CL-6B column (5 cm × 30 cm) that had been equilibrated with 50 mM sodium phosphate buffer (pH 7). After washing with 50 mM sodium phosphate buffer (pH 7), adsorbed proteins were eluted with a linear NaCl gradient from 0 to 1 M. Different fractions were collected and tested for PLA degradation. Active fractions were collected and concentrated via ammonium sulphate precipitation. The obtained enzyme solution was then loaded onto a Macro-prep DEAE column (12.6 mm × 40 mm) that had been equilibrated with 50 mM sodium phosphate buffer (pH 7). Adsorbed proteins were eluted using a linear 0–1 M NaCl gradient in the same buffer. Fractions containing PLA-degrading activity were pooled and concentrated using ammonium sulphate precipitation. Active fractions were loaded onto a Sephacryl S-100 gel filtration column (GE healthcare, Little Chalfont, Buckinghamshire, UK) (2.5 cm × 100 cm) that had been equilibrated with 50 mM sodium phosphate buffer (pH 7). The purified active fractions obtained from the Sephacryl S-100 column were collected and kept at −80 °C.

### 2.7. Protein Determination

Protein contents were determined using the Bradford method with a Bio-Rad dye reagent concentrate. Bovine serum albumin was used as the standard. Aromatic amino acids (phenylalanine, tyrosine, and tryptophan) exhibit an absorption band between 260 and 280 nm. To recover the protein after purification via column chromatography, protein concentrations were estimated by comparison to the absorption at 280 nm of the control tryptophan-containing standard [19].

### 2.8. Sodium Dodecyl Sulphate-Polyacrylamide Gel Electrophoresis

Sodium dodecyl sulphate-polyacrylamide gel electrophoresis (SDS–PAGE) was performed on a 12% gel using a discontinuous system [19]. To visualize protein bands, the gel was stained with Coomassie brilliant blue R-250. A low molecular weight marker was used to estimate the *M*_w_ of the purified proteins.

### 2.9. Effects of pH and Temperature on PLA-Degrading Activity

The optimal pH was determined by mixing PLA-degrading enzyme and PLA-emulsified substrate at different pHs (4–11) and incubating at 50 °C. The pH stability of the PLA-degrading enzyme was determined by dialyzing the enzyme against 50 mM buffer solutions of various pHs (4–11) in seamless cellulose tubing (Daiichi Sankyo, Tokyo, Japan), then measuring the residual activity of the enzyme at pH 7 as described above. The buffer systems used for dialysis were acetate (50 mM, pH 4–5), phosphate (50 mM, pH 6–8), and Na_2_CO_3_–NaHCO_3_ (50 mM, pH 9–11). Dialysis was performed at 4 °C for 24 h. To determine the optimum temperatures for the PLA-degrading enzyme, activities were measured at various temperatures (25–90 °C). The thermal stability of the PLA-degrading enzyme was studied by incubating the samples at various temperatures for 30 min, then measuring residual activities as described above. The PLA-degrading activity measured under standard conditions was considered as 100% active.

### 2.10. Activities of the Purified PLA Depolymerase for Various Substrates

To measure protease activity, a dilute enzyme solution (0.2 mL) was mixed with 1.25 mL of 1.25% casein in phosphate buffer (pH 7) and incubated for 30 min at 37 °C. The reaction was terminated by the addition of 5 mL of 0.19 M trichloroacetic acid (TCA). The reaction mixture was centrifuged, and the soluble peptide content in the supernatant fraction was measured with tyrosine as the reference compound [17]. One unit of protease activity was defined as the amount of enzyme required to release 1 μmol of tyrosine in one minute. Nattokinase activity was measured with a fibrin degradation assay [17]. First, 0.4 mL of 0.72% fibrinogen was placed in a test tube with 0.1 mL of 245 mM phosphate buffer (pH 7) and incubated at 37 °C for 5 min. Then, 0.1 mL of a 20 U/mL thrombin solution was added. The solution was incubated at 37 °C for 10 min, 0.1 mL of diluted enzyme solution was added, and incubation was continued at 37 °C. This solution was mixed after 20 and 40 min of incubation. At 60 min, 0.7 mL of 0.2 M trichloroacetic acid (TCA) was added, and the solution was mixed. The reaction mixture was centrifuged at 15,000× *g* for 10 min. Then, 1 mL of the supernatant was collected, and the absorbance at 275 nm was measured. In this assay, 1 unit (fibrin degradation unit, FU) of enzyme activity is defined as a 0.01 unit increase in the absorbance at 275 nm of the reaction mixture per minute. 

Emulsions of 0.1% (*w*/*v*) triolein, tributyrin, and poly(β-hydroxybutyrate) were prepared with 0.01% (*w*/*v*) Plysurf A210G. The emulsified triolein, tributyrin, or poly(β-hydroxybutyrate) mixtures were incubated with purified enzyme in 10 mM Tris–HCl buffer (pH 9.5) at 37 °C for 30 min. Hydrolysis of the emulsified triolein, tributyrin, or poly(β-hydroxybutyrate) was measured as a decrease in turbidity (absorbance at 630 nm).

## 3. Results and Discussion

### 3.1. Selection of a PLA Depolymerase-Producing Strain

Soil samples were collected from a variety of locations in Taiwan, such as university campuses, house gardens, farmlands, weed fields, plateaus, roadsides, riversides, seasides, and dumping grounds. Small portions of soil sample suspensions were plated onto PLA-emulsified agar medium, and plates were incubated at 30 °C for 5 to 10 days. To screen for microorganisms that produce PLA-degrading enzymes, the medium contained emulsified PLA to induce enzyme production. However, to produce concentrated PLA depolymerase, the nutrient SPP must be added to the agar medium. *P. tamsuii* TKU015, which was stocked in our laboratory, was found to be the most effective PLA-degrading microbe in our culture collection. This microbe formed a large and clear hydrolytic zone on PLA-emulsified agar medium. In following experiments, we investigated the optimum conditions for PLA depolymerase production in *P. tamsuii* TKU015.

### 3.2. Culture Conditions for PLA Depolymerase Production

In our preliminary experiments, both SPP and SSP were used separately as carbon/nitrogen sources to investigate PLA depolymerase production in *P. tamsuii* TKU015. With 0.25% PLA powder, the PLA depolymerase activity of the media after culturing at 30 °C for two days was 0.054 U/mL for media containing 0.25% (*w*/*v*) SPP and 0.017 U/mL for media containing 0.25% (*w*/*v*) SSP. These results indicate that SPP is a better substrate for PLA depolymerase production in TKU015. The effects of various factors, including carbon/nitrogen source concentration, medium volume, medium pH, and culture temperature were consecutively evaluated in single-factor experiments to establish optimal conditions for PLA depolymerase production from *P. tamsuii* TKU015.

#### 3.2.1. Effect of MPS Concentration

**Figure 1:**
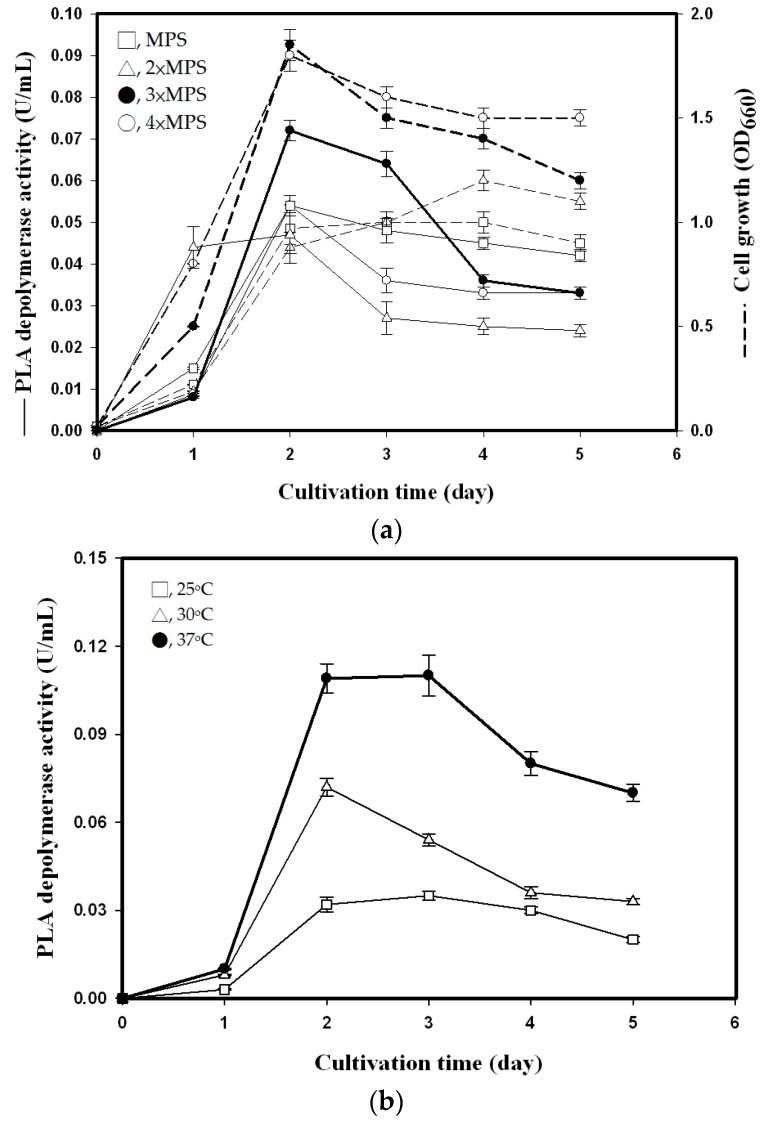
Effects of MPS concentration (**a**) and culture temperature (**b**) on cell growth (dashed line) and PLA depolymerase activity (solid line) of *P. tamsuii* TKU015. All data points are means ± S.D. (standard deviation) of three different experiments performed on different days (each experiment was conducted in triplicate).

Previous studies on biodegradable plastic-degrading enzymes have indicated that medium composition plays a critical role in enzyme production [20,21,22,23]. SPP was first investigated as the carbon/nitrogen source for PLA depolymerase production. To determine the optimal MPS concentration for PLA depolymerase production, bacteria were cultured at 30 °C for five days in media containing one to four times the original concentration of MPS. PLA depolymerase activity in *P. tamsuii* TKU015 and cell growth were greatly increased by switching from MPS to 3×MPS. However, depolymerase activity and growth were not greatly improved by switching from 3×MPS to 4×MPS (Figure 1a). The highest PLA depolymerase activity (0.072 U/mL) was obtained at 3×MPS (Figure 1a). The bacteria grew rapidly for the first two days, and we found that PLA depolymerase activity was closely related to cell growth (Figure 1a). PLA depolymerase activity reached a maximum after the cells reached stationary phase of growth on the second day of incubation. These results indicate that 3×MPS was most suitable for PLA depolymerase production from *P. tamsuii* TKU015 and that the production of PLA depolymerase is growth-dependent. Therefore, 3×MPS was used in subsequent experiments. These results indicated that cell growth is an important factor for enhancing PLA depolymerase production. In addition, *P. tamsuii* TKU015 is a promising source of PLA depolymerase.

#### 3.2.2. Effect of Culture Volume, Initial pH, and Temperature

The effect of culture medium volumes on PLA depolymerase production by *P. tamsuii* TKU015 in 250-mL Erlenmeyer flasks was investigated. TKU015 was inoculated into 3×MPS. PLA depolymerase activity was 0, 0.072, and 0 U/mL for culture volumes of 50, 100, and 150 mL, respectively. Therefore, a 100 mL volume of medium was more suitable for PLA depolymerase production than 50 or 150 mL.

Initial culture pH is also an important factor for depolymerase production, as pH may affect the cell membrane, cell morphology, cell structure, the uptake of various nutrients, and the rate of enzyme production by microorganisms. The optimal medium pH for PLA depolymerase production was found to be 10 before sterilisation. This pH resulted in a PLA depolymerase activity of 0.089 U/mL after cultivation. The degradation of PLA in the medium results in acidification, as the concentration of lactic acid increases when extracellular PLA depolymerase is produced. The alkaline (pH 10) conditions required to neutralize the acid for PLA depolymerase production in *P. tamsuii* TKU015 may be related to the degradation of PLA in the medium.

Incubation temperature is another critical factor for enzyme production in microorganisms. In a preliminary test, *P. tamsuii* TKU015 was cultured at three different temperatures: 4, 25 and 55 °C, representing psychrophilic, mesophilic, and thermophilic conditions, respectively. It was found that *P. tamsuii* TKU015 is a mesophilic bacterium. This result is corroborated by the fact that *P. tamsuii* TKU015 was isolated from a soil sample; soil temperatures are generally below 37 °C. Bacteria were then cultured in conical flasks under the conditions described above (3×MPS, 100 mL, 150 rpm) for five days at various temperatures (25, 30 and 37 °C). The optimal temperature for PLA depolymerase production was found to be 37 °C. After two days of incubation at 37 °C, PLA depolymerase activity in the culture reached 0.109 U/mL (Figure 1b).

#### 3.2.3. Time Course of PLA Depolymerase Production

The use of SPP as the C/N source for PLA depolymerase production was investigated. As shown in Figure 1b, the maximum PLA depolymerase activity (0.109 U/mL) was observed in a 100-mL culture incubated at 37 °C for two days. On the fourth day, PLA depolymerase activity began to decrease. The bacteria grew rapidly during the first two days of culture, and we observed that PLA depolymerase activity was closely related to cell growth. This result indicates that PLA depolymerase production is growth-dependent and that *P. tamsuii* TKU015 is a promising producer of PLA depolymerase. In other published reports, most of the PLA depolymerase-producing strains discovered to date are produced by the *Amycolatopsis* genus [5,11,12,13,14,15,24] and several fungal strains [23,25,26,27,28]. However, PLA degradation by fungi is very slow. In contrast to studies regarding PLA degradation by *Amycolatopsis* spp. and fungi, reports of PLA degradation by bacteria are rare. Reports of the purification of PLA-degrading enzymes from bacteria are rare as well. Furthermore, most of the studies on PLA depolymerase-producing strains examined depolymerase production in synthetic medium. In this study, *P. tamsuii* TKU015 adjusted to the imposed culture conditions and were able to use fish waste SPP as a C/N source for PLA depolymerase production.

### 3.3. Purification of the Enzyme

**Figure 2:**
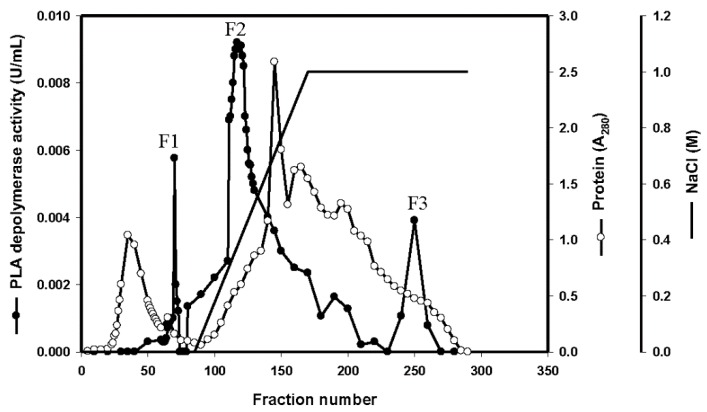
Elution profile of TKU015 PLA depolymerase from DEAE-Sepharose CL-6B.

**Figure 3:**
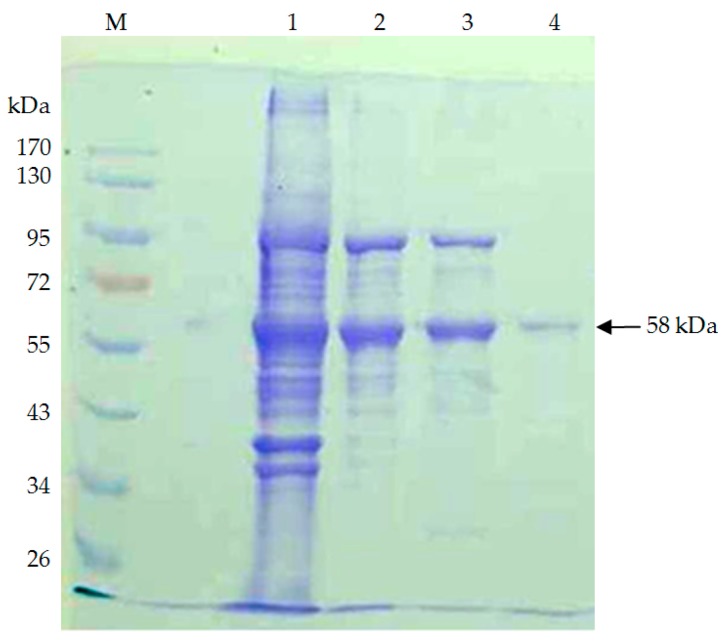
Sodium dodecyl sulphate polyacrylamide gel electrophoresis (SDS–PAGE) analysis of PLA depolymerase produced by *P. tamsuii* TKU015. Lanes: M, molecular markers (170, 130, 95, 72, 55, 43, 34, and 26 kDa); (1) crude enzyme; (2) adsorbed PLA depolymerase fractions after DEAE-Sepharose CL-6B chromatography; (3) adsorbed PLA depolymerase fractions after Macro-prep DEAE chromatography; (4) PLA depolymerase fractions after Sephacryl S-100.

The production of PLA depolymerase by strain TKU015 was investigated over two days of cultivation in the most suitable medium. Extracellular PLA depolymerase was purified from the culture supernatant of *P. tamsuii* TKU015 using a series of purification procedures. During the DEAE-Sepharose CL-6B chromatography step, TKU015 PLA depolymerase was eluted with a linear 0–1 M NaCl gradient, generating three peaks with PLA-degrading activity (Figure 2). Fractions corresponding to each peak (defined as F1, F2, and F3) were pooled separately and dialyzed. We confirmed that protein elution with this gradient was not complete. However, of the eluted peaks, F2 was the major component, exhibiting a high protein concentration and high PLA-degrading activity. Therefore, we subjected F2 to further purification with Macro-prep DEAE chromatography. The adsorbed PLA depolymerase fractions were pooled and concentrated using ammonium sulphate precipitation. After dialysis against 50 mM phosphate buffer (pH 7), purification was performed using a Sephacryl S-100 gel filtration column. This procedure resulted in approximately 10.8 mg of TKU015 PLA depolymerase (Table 1). A summary of the purification process is presented in Table 1. In combination, the purification steps resulted in approximately 9.3-fold purification of TKU015 PLA depolymerase. Overall TKU015 PLA depolymerase activity yield was 8%, with a specific activity of 0.028 U/mg. The low activity and low protein recovery might be due to incomplete elution of the adsorbed protein at the DEAE chromatography step as well as loss of active protein during the concentration process. The molecular mass of TKU015 PLA depolymerase was approximately 58 kDa; this mass was confirmed by SDS-PAGE analysis (Figure 3) and corresponded to the gel-filtration chromatography results. The molecular mass of TKU015 PLA depolymerase (58 kDa) was different from the masses of PLA depolymerases from *Amycolatopsis* spp., which have low apparent molecular masses ranging from 18 to 24 kDa [13,24].

### 3.4. Effects of pH and Temperature

**Figure 4:**
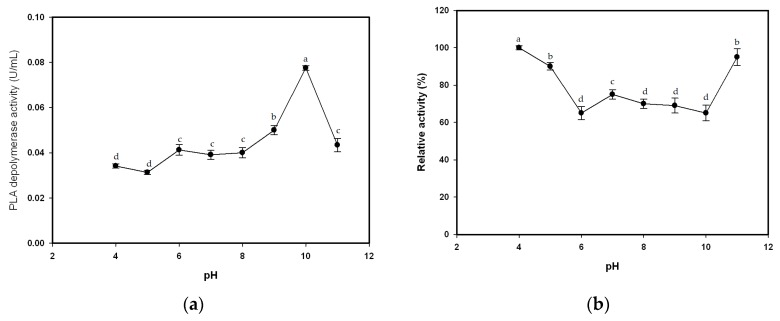

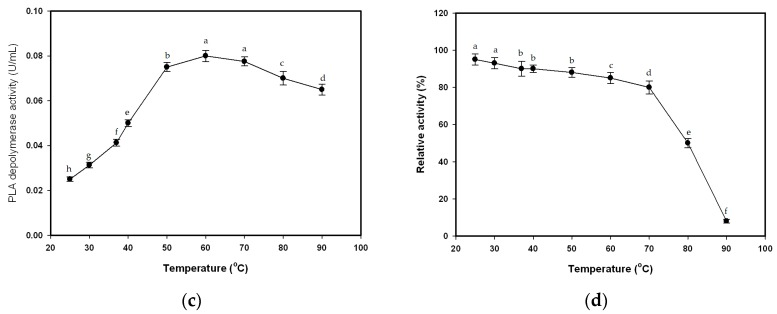
Effects of pH and temperature on the activity and stability of the PLA depolymerase from *P. tamsuii* TKU015. (**a**) Optimum pH; (**b**) pH stability; (**c**) optimum temperature; (**d**) thermal stability. All data points are means ± S.D. of three different experiments performed on different days (each experiment was conducted in triplicate). Means with different letters are significantly different at *p* < 0.05.

The degradation of emulsified PLA by purified enzyme was assayed under standard conditions and pH values ranging from 4 to 11. In the pH 5–9 range, the PLA-degrading activity of the enzyme increased slightly with increasing pH (Figure 4a). The pH activity profiles of TKU015 PLA depolymerase exhibited maximum activity at pH 10 (Figure 4a). In contrast, enzyme activity is lower under acidic conditions (pH < 5; Figure 4a). The optimum pH (pH 10) for TKU015 PLA depolymerase activity was similar to that of most bacterial PLA depolymerases, which display optimum activities at alkaline pH values ranging from 8.5 to 10.5 [1,13,24]. The pH stability profiles of TKU015 PLA depolymerase were determined by measuring residual enzyme activities at pH 10 after incubation at pHs ranging from 4 to 11 at 37 °C for 60 min. As shown in Figure 4b, the stability of TKU015 PLA depolymerase decreased when incubated at pHs between 6 and 10. However, the depolymerase still retained more than 65% of its initial activity in this pH range.

The effect of temperature on the activity of TKU015 PLA depolymerase was also studied. The optimum temperature for TKU015 PLA depolymerase activity was 60 °C (Figure 4c). To examine the thermal stability of TKU015 PLA depolymerase, the enzyme solution in 50 mM phosphate buffer (pH 7) was incubated for 60 min at various temperatures, and residual activity was measured. Less than 10% of the initial enzyme activity could be detected after incubation at 90 °C (Figure 4d). TKU015 PLA depolymerase was relatively stable from 25 to 70 °C and had 50% of its activity at 80 °C, but it was inactivated at 90 °C (Figure 4d). The thermal stability of TKU015 PLA depolymerase is different from that of most bacterial PLA depolymerases, which are completely inactivated at 70 °C [1,13,24]. 

### 3.5. Substrate Specificity

The substrate specificity of TKU015 PLA depolymerase was tested to determine the hydrolytic activity of the enzyme against several representative protease, nattokinase, esterase, and lipase substrates. As shown in Table 2, TKU015 PLA depolymerase showed remarkable specificity for fibrinogen and tributyrin. However, the enzyme did not exhibit significant hydrolytic activity on casein, triolein, and poly(β-hydroxybutyrate) (Table 2). Thus, TKU015 PLA depolymerase exhibited nattokinase and esterase activities but did not display protease, lipase, and PHB hydrolysis activities.

The degradation of PLA has been reported to proceed mainly via protease-like enzymes. Proteinase K has been previously recognized as a representative PLA-degrading enzyme [9,10,11,12,13,14,15,19,26]. It can hydrolyze high-molecular-weight PLA and casein with the properties as alkaline serine proteases. Compared with proteinase K, TKU015 PLA depolymerase showed no proteolytic activity against casein, but had the degrading activity against PLA. It has also been reported that lipases from *Pseudomonas* sp. strain DS04-T [1] and *R. delemer* [26], and the polyester polyurethane-degrading enzyme from *C. acidovorans* strain TB-35 [9] have PLA-degrading activities. As shown in Table 2, TKU015 PLA depolymerase showed no activity against triolein (a representative substrate for lipase), which was the different characteristic of the lipase from *R. delemer* [26]. TKU015 PLA depolymerase hydrolyzed tributyrin (a substrate for esterase), which was the similar characteristic of the polyester polyurethane-degrading enzyme from *C. acidovorans* strain TB-35 [9] for PLA degradation. However, the properties of TKU015 PLA depolymerase were different from the polyester polyurethane-degrading enzyme from *C. acidovorans* strain TB-35 [9]. Taken together, TKU015 PLA depolymerase had no protease and lipase activities and is different from previously reported PLA depolymerases. TKU015 PLA depolymerase may be more adaptable to catalyze the hydrolysis of the ester bond between lactate units. Thus, TKU015 PLA depolymerase is a novel PLA-degrading enzyme.

## 4. Conclusions

In this study, *P. tamsuii* TKU015 is a potent PLA depolymerase-producing strain, it can utilize SPP and recycled PLA plastic waste as the carbon/nitrogen sources to produce PLA depolymerase with high productivity. This is different from most other reported PLA depolymerase-producing strains which use synthetic medium. The utilization of inexpensive culture medium and a practical fermentation process can produce a more economically competitive PLA-degrading enzyme for recycling of fishery wastes and PLA wastes in large amounts. In addition, a unique PLA depolymerase was purified from the culture supernatant of TKU015 and the biochemical properties of the purified PLA depolymerase have also been characterized. These findings appear useful for further applications of TKU015 PLA depolymerase in biodegradation of plastic materials.

## Figures and Tables

**Table 1:** Purification of PLA depolymerase from *P. tamsuii* TKU015 ^a^.

Step	Total Protein (mg)	Total Activity (U)	Specific Activity (U/mg Protein)	Purification Fold	Yield (%)
Culture supernatant	1425.0	3.6	0.003	1.0	100
(NH_4_)_2_SO_4_ ppt	220.1	0.9	0.004	1.3	25
DEAE-Sepharose	42.1	0.4	0.010	3.3	11
Macro-prep DEAE	35.5	0.4	0.011	3.7	11
Sephacryl S-100	10.8	0.3	0.028	9.3	8

^a^ *P. tamsuii* TKU015 was grown in 100 mL of liquid medium in an Erlenmeyer flask (250 mL) containing 3×MPS, 0.1% K_2_HPO_4_, and 0.05% MgSO_4_·7H_2_O in a shaking incubator for two days at 37 °C.

**Table 2:** Substrate specificities of TKU015 PLA depolymerase for the hydrolysis of several representative substrates.

Substrate	Hydrolysis Activity (U/mg)
Casein	<0.10 ± 0.002 ^c^
Fibrinogen	7.22 ± 0.25 ^a^
Poly(β-hydroxybutyrate)	<0.10 ± 0.001 ^c^
Tributyrin	3.51 ± 0.16 ^b^
Triolein	<0.10 ± 0.001 ^c^

All data are expressed as mean ± S.D. from three different experiments (each experiment was conducted in triplicate). Data with different letters are significantly different at *p* < 0.05.
